# NK Cell Function Regulation by TGF-β-Induced Epigenetic Mechanisms

**DOI:** 10.3389/fimmu.2020.00311

**Published:** 2020-02-25

**Authors:** Stefano Regis, Alessandra Dondero, Fabio Caliendo, Cristina Bottino, Roberta Castriconi

**Affiliations:** ^1^Laboratory of Clinical and Experimental Immunology, IRCCS Istituto Giannina Gaslini, Genoa, Italy; ^2^Department of Experimental Medicine, University of Genoa, Genoa, Italy; ^3^Department of Biological Engineering, Synthetic Biology Center, Massachusetts Institute of Technology, Cambridge, MA, United States; ^4^Centre of Excellence for Biomedical Research, CEBR, University of Genoa, Genoa, Italy

**Keywords:** NK cells, TGF-β, epigenetic, transcription factors, microRNAs

## Abstract

TGF-β is a potent immunosuppressive cytokine that severely affects the function of NK cells. Tumor cells can take advantage of this ability, enriching their surrounding microenvironment with TGF-β. TGF-β can alter the expression of effector molecules and of activating and chemokine receptors, influence metabolism, induce the NK cell conversion toward the less cytolytic ILC1s. These and other changes possibly occur by the induction of complex gene expression programs, involving epigenetic mechanisms. While most of these programs are at present unexplored, the role of certain transcription factors, microRNAs and chromatin changes determined by TGF-β in NK cells start to be elucidated in human and/or mouse NK cells. The deep understanding of these mechanisms will be useful to design therapies contributing to restore the full NK function.

## Introduction

NK cells are innate lymphocytes able to recognize and kill virus-infected and tumor cells. They are equipped with a set of inhibitory and activating receptors, whose integrated signaling selectively directs their activity (1). While inhibitory receptors bind molecules such as HLA class I or immune checkpoint ligands, activating receptors interact with stress molecules induced or upregulated by tumor transformation or viral infection. Several cytokines, like IL-2, IL-12, IL-15, and IL-18, influence NK cell activity. Stimulated NK cells can, in turn, produce other cytokines, including IFN-γ and TNF-α (1). Traditionally, two populations of NK cells are described, CD56^bright^, prevailing in secondary lymphoid organs, producing large amounts of cytokines, and CD56^dim^, abundant in peripheral blood, characterized by high cytotoxic potential. However, recent views consider the spectrum of NK cell diversity quite wider (2–4). Moreover, NK cells represent the cytolytic members of a large, innate lymphoid cell family (ILC) that includes ILC1, poor cytolytic cells sharing with NK cells the ability to produce IFN-γ, ILC2, secreting IL-5 and IL-13, and ILC3, producing IL-7 and IL-22 (5, 6).

The activity of NK cells can be strongly affected by TGF-β, an immunomodulatory cytokine with a prominent role in both innate and adaptive immune responses (7, 8). In particular, TGF-β is a negative regulator of IFN-γ production (9) and decreases the surface level of the activating receptors NKG2D and NKp30, reducing the cytotoxic ability of NK cells (10) and impairing their antitumor function. Accordingly, mice lacking the expression of the TGF-β receptor 2 (TGFBR2) in their NK cells show improved suppression of metastases compared to control mice (11). TGF-β, also alters the surface expression of the chemokine receptors CXCR3, CXCR4, CX3CR1, with a possible impact on NK cell migration and recruitment (12). Surprisingly, some of the modulatory effects of TGF-β are potentiated by IL-18, via p38/MAPK pathway (13). The negative regulatory role of TGF-β also extends to different metabolic pathways including glycolysis and mitochondrial processes (14, 15).

TGF-β acts on target cells via specific receptors composed of TGFBR1 and TGFBR2 subunits, which can transduce the signal phosphorylating SMAD2 and SMAD3 proteins. These molecules bind to SMAD4 forming heterotrimeric transcriptional complexes that accumulate in the nucleus, where they activate or repress the transcription of sets of target genes. Other SMAD-independent non-canonical pathways proceed via PI3K and MAPK (16).

TGF-β signaling has been associated with epigenetic alterations (16), i.e., reversible and heritable changes not associated with DNA sequence mutation. These include DNA methylation, post-translational modification of chromatin histones as well as changes in the expression of transcription factors and noncoding RNAs (17).

Several epigenetic modifications contributing to the deep influence of TGF-β on the NK cell function, including chromatin remodeling, induction or repression of transcription factors, and post-transcriptional regulation of gene expression by miRNAs, have been described, and are here reviewed.

## Transcription Factors and Chromatin Remodeling

**Figure 1:**
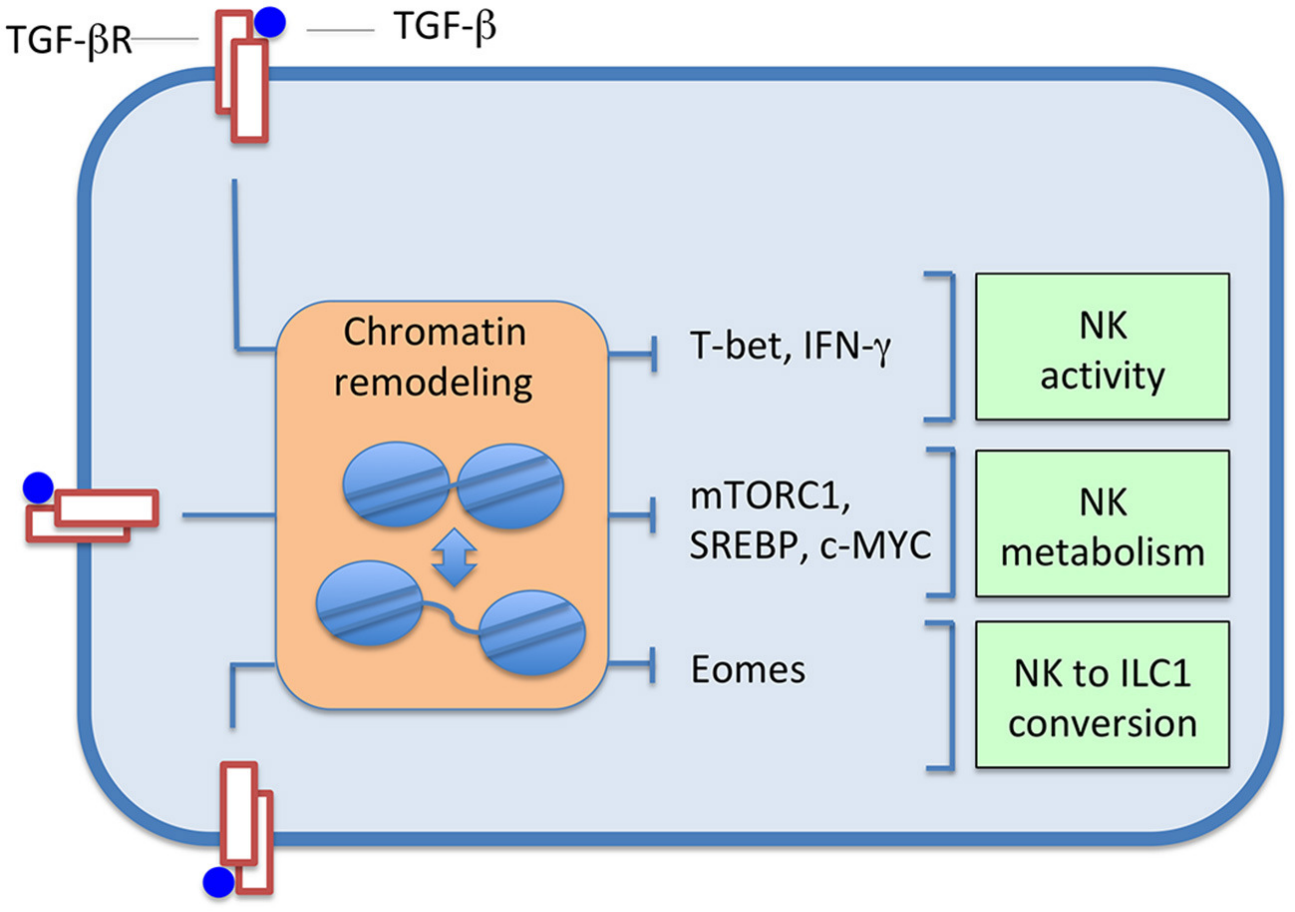
TGF-β induces changes in the expression of several transcription factors, and, consequently, in sets of controlled genes. Effects, often accompanied by chromatin alterations, range from inhibition of NK cell activity, to depression of cell metabolism, up to the induced conversion of NK cells to ILC1s.

TGF-β affects NK cell function inducing relevant transcription programs, which can involve events of chromatin remodeling, through the action of various transcription factors; the landscape of these changes is presumably wide and mostly unexplored. Studies have been performed on the SMAD3-dependent regulation of T-bet and IFN-γ expression, on NK cell metabolism and conversion of NK cells to ILC1s (Figure 1).

### T-bet-IFN-γ Pathway

Pro-inflammatory cytokines positively regulate the production of IFN-γ and the expression of the transcription factor T-bet, which, as the other T-box protein Eomes, has a relevant role in development and function of NK cells (18). Acting as an immunosuppressive cytokine, TGF-β antagonizes the effects of pro-inflammatory cytokines, down-regulating T-bet and IFN-γ via SMAD3 (9, 19). In line with this evidence, SMAD3-deficient mouse NK cells produced more IFN-γ (9), and TGFBR2-deficient mouse NK cells stimulated with IL-15 showed increased T-bet expression (11). Interestingly, chromatin remodeling has been associated with the T-bet-dependent regulation of IFN-γ gene expression in CD4 T cells polarizing toward T_H_1 cells (20). Moreover, it has been shown that T-bet, besides its ability to interact with the IFN-γ promoter and with several enhancers, can recruit histone-modifying complexes inducing epigenetic modifications at the IFN-γ locus (20, 21). In response to activating receptor engagement, CD56^dim^ human NK cells showed increased expression of T-bet and IFN-γ, compared to CD56^bright^ NK cells. High IFN-γ production was accompanied by a reduced CpG methylation pattern and histone modifications of the IFN-γ gene promoter (22). Moreover, epigenetic remodeling of the *IFNG* conserved non-coding sequence (CNS) 1, located upstream of the human IFN-γ promoter, has been documented in NKG2C^hi^ “adaptive” NK cells, which expand in CMV seropositive individuals (23).

Interestingly, “TGF-β-imprinted” NK cells, produced by prolonged IL-2 and TGF-β stimulation of activated NK cells, contrarily to acutely TGF-β-treated NK cells, exhibited a pro-inflammatory phenotype, with abundant production of IFN-γ and TNF-α persisting after removal of TGF-β (24). The TGF-β pathway was altered in these cells, as indicated by the down-regulation of SMAD3, whose locus showed reduced chromatin accessibility, and of T-bet.

Of note, TGF-β can inhibit IFN-γ expression both by a T-bet dependent and a T-bet independent manner (9). In the latter context, Tang et al. (25) reported that SMAD3 suppresses transcription of IFN-γ via E4BP4, which is an important transcription factor for the NK cell lineage commitment (26).

From a more general perspective, the intimate link between T-bet and chromatin modifications is underlined by a recent study exploring chromatin and gene expression changes associated with viral infection in mouse CD8 T cells. The authors reported that T-box and Runx genes have been shown to act as critical mediators of chromatin remodeling during CD8 T cell activation (27).

### NK Cell Metabolism: mTORC1, SREBP, and c-MYC

TGF-β can sensibly reduce NK cell metabolism affecting glycolysis and oxidative phosphorylation, which are considerably increased in cytokine-stimulated NK cells and support effector functions (11). The serine/threonine kinase mTOR is part of the mTORC1 complex, a master regulator of various metabolic processes in different cell types. mTORC1 activity is important for activation-induced metabolic and functional responses in NK cells (14). TGF-β was found to inhibit mTOR-dependent activities in NK stimulated by IL-15 (11). Moreover, mouse TGFBR2-defective NK cells stimulated with IL-15 showed increased mTOR-dependent activities. mTORC1 also contributed to the regulation of the SREBP (28) and c-MYC (29) transcription factors, which are key controllers of glycolysis and oxidative phosphorylation in cytokine-stimulated NK cells (14).

In humans, it has been reported that TGF-β, acting by the canonical pathway, significantly decreases the rate of IL-2-induced mitochondrial metabolism, and inhibits the glycolysis in IL-2 stimulated human NK cells independently by mTORC1 (14, 30).

### NK-ILC1 Cell Conversion

The potency of TGF-β action is further highlighted by its ability to induce the conversion of NK cells to ILC1s (31, 32). Although NK cells and ILC1s are both IFN-γ producers and functionally dependent on T-bet (5), they are developmentally distinct. ILC1s are not found in blood and lymphoid organs, having instead tissue-resident features (33) and being weakly cytolytic (5). Moreover, while mature NK cells express Eomes, ILC1s do not.

Gao et al. (31) reported that, in the tumor microenvironment, TGF-β can induce the conversion of mouse NK cells to an NK-ILC1 intermediate cell type (intILC1s) and, finally, to ILC1s; the switch is accompanied by the down-regulation of the transcription factor Eomes. Compared to NK cells, intILC1s and ILC1s are unable to control tumor growth and metastasis. Therefore, TGF-β, inducing the conversion, inhibits cancer immunosurveillance by a new mechanism of immune evasion. Cortez et al. (32) generated SMAD4-deficient NK cells, which showed features of ILC1s, had impaired effector functions and were unable to control tumor metastasis, thus indicating that SMAD4 is a negative regulator of the NK-ILC1s conversion. In particular, SMAD4 was shown to hamper the conversion of NK cells to ILC1s inhibiting a non-canonical TGFBR1-mediated pathway.

As stated by the RNAseq-based transcriptome data obtained by NKs, intILC1s, and ILC1s cell populations (31), the NK to ILC1 conversion induced by TGF-β involves sets of genes, presumably governed by still undefined, complex, transcriptional and epigenetic programs. Given the reduction of Eomes expression during cell conversion and its potential role as chromatin remodeler, we can speculate that it may have a relevant role in this event of cell plasticity. In a different context, similar considerations can be done for T-bet, which is reduced by TGF-β in human liver resident NK cells, this reduction being fundamental for the maintenance of the Eomes^hi^ T-bet^lo^ phenotype of this subtype of NK cells (4).

Notably, together with IL-23, TGF-β guides the conversion ILC1 toward ILC3, which can occur in mucosal tissues (34). TGF-β increases the expression of the transcription factors T-bet and Aiolos. Interestingly, Aiolos, like the other IKZF members, can suppress target gene expression altering chromatin accessibility via histones deacetylation (35). By this mechanism, it suppresses the ILC3 specific genes IL-22 and RORγt, promoting the ILC3-ILC1 conversion (34).

## miRNAs

**Figure 2:**
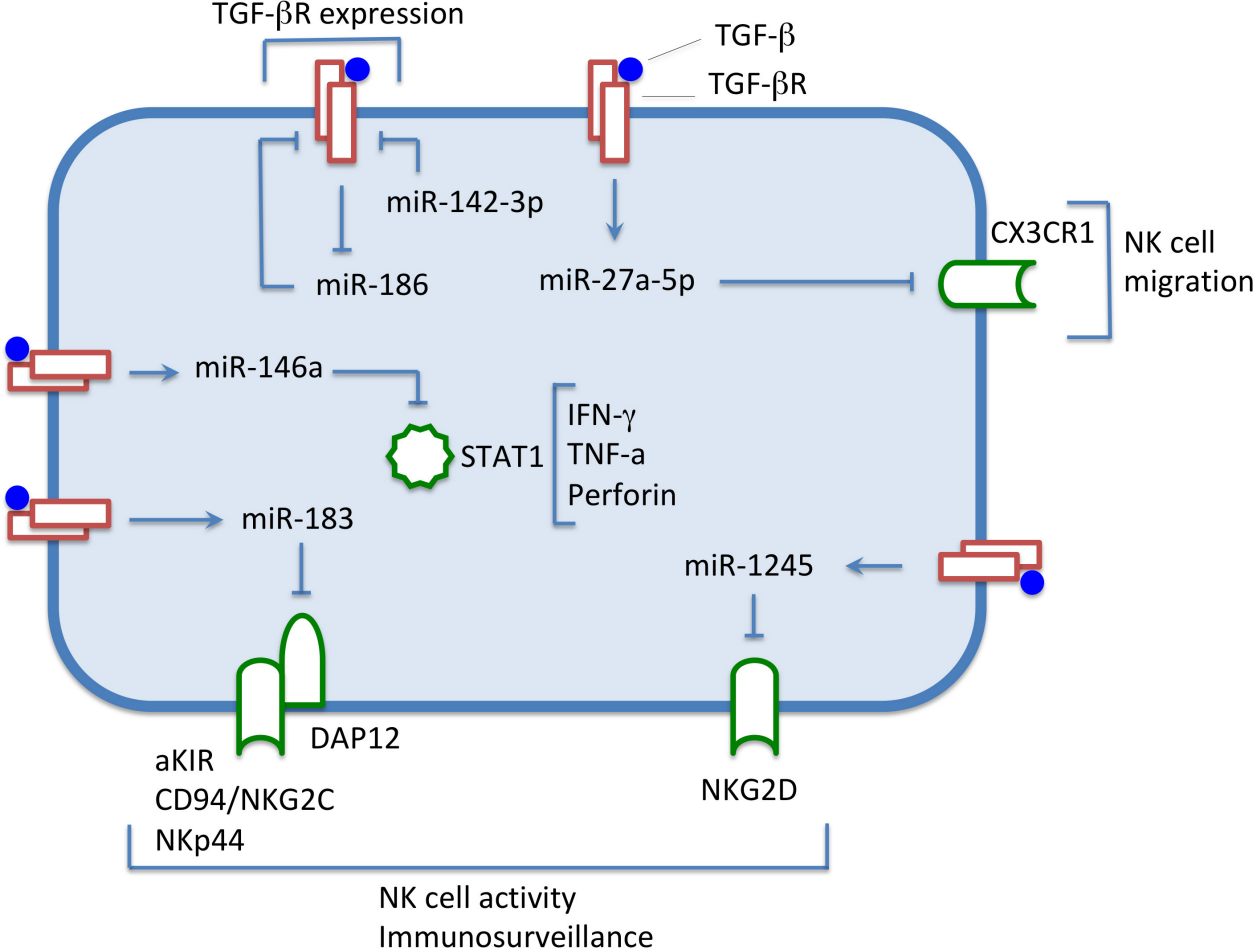
Under the influence of TGF-β, levels of expression of some miRNAs are increased, down-regulating the expression of surface molecules and transcription factors involved in NK cell activity. Conversely, miRNA-186 level decreases, thus preventing the down-regulation of the TGF-β receptor expression, and maintaining the NK cells responsiveness to the immunomodulatory cytokine. miR-142-3p down-regulates TGFBR1, thus modulating the TGF-β signaling.

Besides chromatin remodeling and transcription factor manipulation, TGF-β can affect the NK cell function acting on the miRNA machinery, exploiting its capacity to influence gene expression at the post-transcriptional level. Distinct miRNA-containing pathways deregulating the expression of receptors or transcription factors have been described (Figure 2) (36).

### miR-1245

NKG2D is a C-type lectin-like activating receptor expressed on NK and CD8 T cells, which promotes the elimination of transformed and pathogen-infected cells. In humans, it recognizes stress-inducible ligands belonging to the MIC (MICA and MICB) and ULBP (ULBP1-6) families (37).

The surface expression of NKG2D is significantly down-regulated in NK cells by TGF-β1 or TGF-β2 (10). Espinoza et al. (38) reported that TGF-β1 causes an increase of miR-1245 in human NK cells. Overexpression of miR-1245 induced a down-regulation of NKG2D at the cell surface without affecting the other activating receptors NKp30, NKp44, and NKp46. Moreover, NK cells overexpressing miR-1245 had lower cytotoxicity against target cells expressing NKG2D ligands. MiR-1245 was shown to directly target and down-regulate the NKG2D mRNA. Interestingly, a polymorphism in the NKG2D 3′-UTR region occurs in the region complementary to the miR-1245 seed region (39). NK cells carrying this polymorphism exhibit a reduced TGF-β-mediated modulation of NKG2D activity, which can be due to less efficient targeting of the miRNA. Importantly, the NKG2D variants seem to influence immunosurveillance capability and risk of cancer development (40).

### miR-183

DAP12 (also known as KARAP or TYROBP) is a 12 kDa transmembrane protein containing an immune tyrosine-based activation motif (ITAM) in its cytoplasmic domain (41). DAP12 is a signaling molecule associating with different activating receptors, including the activating KIRs, CD94/NKG2C, and NKp44, the latter expressed by NK cells upon activation and by a subset of ILC3 (42).

Donatelli et al. (43) reported that TGF-β1 down-regulates the DAP12 level in human NK cells. In particular, it up-regulated miR-183, which was shown to directly target DAP12 mRNA. The NK92 cell line overexpressing miR-183 had reduced DAP12, decreased NKp44 surface levels, and was less efficient in killing the Raji Burkitt lymphoma cell line.

Interestingly, NK cells infiltrating lung cancers were shown to have a diminished DAP12 expression (43). Recently, a mechanism of immune evasion has been described in human lung cancer cells (44). TGF-β appears to act simultaneously on NK cells and tumor cells via miR-183, with the result to inhibit the NK cells' anti-tumor activity by down-regulating DAP12, in NK cells, and NKG2D ligands (MICA and MICB), in cancer cells.

### miR-27a-5p

CX_3_CR1 is a chemokine receptor expressed by different immune cell types, including NK cells, which binds CX_3_CL1 (also known as fractalkine). CX_3_CR1 drives, with other chemokine receptors, NK cells localization at a steady state in peripheral tissues, and promotes their migration under inflammatory conditions (45). Moreover, CX_3_CR1, CXCR4, and S1P5 regulate NK cells homing and egress from the bone marrow (46, 47).

It has been shown that TGF-β1 released by neuroblastoma tumor cells down-regulated the surface expression of CX_3_CR1 in human NK cells (12). Afterward, TGF-β1 was found to up-regulate in NK cells the miR-23a-27a-24-2 cluster, encoding, among others, miR-27a-5p, that has been demonstrated to target CX_3_CR1 (48). In agreement with this finding (12), NK cells cultured in the presence of neuroblastoma cells exhibited a significant inverse correlation between miR-27a-5p and CX_3_CR1 mRNA expression (48). Notably, an unusual CX_3_CR1^low/neg^ phenotype has been described in circulating CD56^dim^ NK cells and in NKp46^+^ bone marrow resident NK cells from high-risk NB patients (12).

### miR-186

Neviani et al. (49) reported that miR-186 down-regulates TGFBR1 and TGFBR2 preventing the TGF-β-dependent inhibition of human NK cells. MiR-186 was found to be down-regulated in TGF-β-treated NK cells as well as in neuroblastoma cells. Importantly, by receiving miR-186, carried by NK exosomes, neuroblastoma cells down-regulated the oncogenic proteins MYCN and AURKA. Therefore, delivery of miR-186 to neuroblastoma and NK cells may hamper both tumorigenic potential and inhibition of NK cells.

### miR-142

MiR-142 is a critical regulator of ILC biology. It is induced by IL-15 and mediates modulation of TGFBR1. miR-142^−/−^ knockout mice showed increased TGF-β signaling compared to normal controls, as indicated by the augmented expression of TGFBR1 and functionally related genes in NK/ILC1 cells from spleen and bone marrow (50). Interestingly, miR-142^−/−^ mice presented an altered number and functionality of NK cells, with increased susceptibility to cytomegalovirus infection. On the contrary, an increase of ILC1-like cells was observed, possibly related to the increased TGF-β signaling and NK-ILC1 conversion.

### miR-146a

Xu et al. (51) reported that human miR-146a, which is up-regulated by TGF-β in NK cells, targets STAT1, a transcription factor involved in interferons signal transduction, which contributes to NK cell cytotoxicity (52). miR-146 overexpression lowered the production of effector molecules as IFN-γ, TNF-α, and perforin. Moreover, the expression level of miR-146a negatively correlated with NK cell-mediated cytotoxicity (51).

## Concluding Remarks

TGF-β can exert its immunosuppressive action influencing NK cells at different levels. Acting by transcription factors, TGF-β can hamper NK cell function inhibiting the T-bet**-**IFN-γ pathway and negatively influences cell metabolism, thus hampering cell activation. Using miRNAs, TGF-β down-regulates activating receptors, receptor-associated signaling molecules, and chemokine receptors, thus inhibiting NK cell cytotoxicity and recruitment in tissues. Acting by still undefined combined transcriptional and epigenetic programs, TGF-β converts NK cells to ILC1, which are less cytolytic against tumor cells. Moreover, we can speculate that long non-coding RNA (lncRNAs), which are known to be involved in TGF-β-driven fibrosis, epithelial-mesenchymal transition and cancer progression (53, 54), may represent additional mediators of still undefined TGF-β-dependent immune-modulatory functions on NK cells.

Studying how TGF-β alters the NK/ILC biology through epigenetic mechanisms could be an important area of investigation to understand its functions in the tumor microenvironment and in other diseases containing TGF-β rich milieus, such as fibrosis. In this context, it will be also important the development of molecular approaches aimed to control the TGF-β signaling. Several strategies have been recently elaborated for the therapeutic inhibition of the TGF-β pathway, particularly to improve the antitumor immune responses. Combination therapy with an inhibitor of TGFβ type I receptor (TGFβR1) kinase, galunisertib, anti-GD2 antibody and *ex vivo* activated NK cells, has been used by Tran et al. (55) in a mouse model of neuroblastoma with positive results. Genetically modified SMAD3-silenced human NK-92 cells inhibited cancer progression in two xenograft mouse models with human hepatoma and melanoma (56). NK cells genetically modified to express a truncated TGFBR2 receptor fused to the DAP12 activation domain exhibited higher cytotoxic activity against neuroblastoma (57). A deeper knowledge of the mechanisms by which TGF-β acts on NK cells will be useful to improve therapeutic strategies aimed to efficiently restore NK cell activity or to increase the NK cell pool for an effective antitumor response.

## Author Contributions

All authors contributed to the writing of the manuscript.

### Conflict of Interest

The authors declare that the research was conducted in the absence of any commercial or financial relationships that could be construed as a potential conflict of interest.

## References

[1] Björkström NK Ljunggren H-G Michaëlsson J. Emerging insights into natural killer cells in human peripheral tissues. Nat Rev Immunol. (2016) 16:310–20. 10.1038/nri.2016.34 27121652

[2] Freud AG Mundy-Bosse BL Yu J Caligiuri MA. The broad spectrum of human natural killer cell diversity. Immunity. (2017) 47:820–33. 10.1016/j.immuni.2017.10.008 29166586 PMC5728700

[3] Crinier A Milpied P Escalière B Piperoglou C Galluso J Balsamo A. High-dimensional single-cell analysis identifies organ-specific signatures and conserved NK cell subsets in humans and mice. Immunity. (2018) 49:971–86.e5. 10.1016/j.immuni.2018.09.009 30413361 PMC6269138

[4] Harmon C Jameson G Almuaili D Houlihan DD Hoti E Geoghegan J. Liver-derived TGF-β maintains the EomeshiTbetlo phenotype of liver resident natural killer cells. Front Immunol. (2019) 10:1502. 10.3389/fimmu.2019.01502 31333651 PMC6616151

[5] Spits H Bernink JH Lanier L. NK cells and type 1 innate lymphoid cells: partners in host defense. Nat Immunol. (2016) 17:758–64. 10.1038/ni.3482 27328005

[6] Spits H Artis D Colonna M Diefenbach A Di Santo JP Eberl G. Innate lymphoid cells–a proposal for uniform nomenclature. Nat Rev Immunol. (2013) 13:145–9. 10.1038/nri3365 23348417

[7] Batlle E Massagué J. Transforming growth factor-β signaling in immunity and cancer. Immunity. (2019) 50:924–40. 10.1016/j.immuni.2019.03.024 30995507 PMC7507121

[8] David CJ Massagué J Contextual determinants of TGFβ action in development, immunity and cancer. Nat Rev Mol Cell Biol. (2018) 19:419–35. 10.1038/s41580-018-0007-0 29643418 PMC7457231

[9] Yu J Wei M Becknell B Trotta R Liu S Boyd Z. Pro- and antiinflammatory cytokine signaling: reciprocal antagonism regulates interferon-gamma production by human natural killer cells. Immunity. (2006) 24:575–90. 10.1016/j.immuni.2006.03.016 16713975

[10] Castriconi R Cantoni C Della Chiesa M Vitale M Marcenaro E Conte R. Transforming growth factor beta 1 inhibits expression of NKp30 and NKG2D receptors: consequences for the NK-mediated killing of dendritic cells. Proc Natl Acad Sci USA. (2003) 100:4120–5. 10.1073/pnas.0730640100 12646700 PMC153058

[11] Viel S Marçais A Guimaraes FS-F Loftus R Rabilloud J Grau M. TGF-β inhibits the activation and functions of NK cells by repressing the mTOR pathway. Sci Signal. (2016) 9:ra19. 10.1126/scisignal.aad1884 26884601

[12] Castriconi R Dondero A Bellora F Moretta L Castellano A Locatelli F. Neuroblastoma-derived TGF-β1 modulates the chemokine receptor repertoire of human resting NK cells. J Immunol. (2013) 190:5321–8. 10.4049/jimmunol.1202693 23576682

[13] Casu B Dondero A Regis S Caliendo F Petretto A Bartolucci M. Novel immunoregulatory functions of IL-18, an accomplice of TGF-β1. Cancers. (2019) 11:E75. 10.3390/cancers11010075 30641867 PMC6356463

[14] O'Brien KL Finlay DK. Immunometabolism and natural killer cell responses. Nat Rev Immunol. (2019) 19:282–90. 10.1038/s41577-019-0139-2 30808985

[15] Viel S Besson L Marotel M Walzer T Marçais A. Regulation of mTOR, metabolic fitness, and effector functions by cytokines in natural killer cells. Cancers. (2017) 9:E132. 10.3390/cancers9100132 28956813 PMC5664071

[16] Massagué J. TGFβ signalling in context. Nat Rev Mol Cell Biol. (2012) 13:616–30. 10.1038/nrm3434 22992590 PMC4027049

[17] Bugide S Janostiak R Wajapeyee N. Epigenetic mechanisms dictating eradication of cancer by natural killer cells. Trends Cancer. (2018) 4:553–66. 10.1016/j.trecan.2018.06.004 30064663 PMC6085095

[18] Zhang J Marotel M Fauteux-Daniel S Mathieu A-L Viel S Marçais A. T-bet and Eomes govern differentiation and function of mouse and human NK cells and ILC1. Eur J Immunol. (2018) 48:738–50. 10.1002/eji.201747299 29424438

[19] Trotta R Col JD Yu J Ciarlariello D Thomas B Zhang X. TGF-β utilizes SMAD3 to inhibit CD16-mediated IFN-γ production and antibody-dependent cellular cytotoxicity in human NK cells. J Immunol. (2008) 181:3784–92. 10.4049/jimmunol.181.6.3784 18768831 PMC2924753

[20] Wilson CB Rowell E Sekimata M. Epigenetic control of T-helper-cell differentiation. Nat Rev Immunol. (2009) 9:91–105. 10.1038/nri2487 19151746

[21] Miller SA Weinmann AS. Molecular mechanisms by which T-bet regulates T-helper cell commitment. Immunol Rev. (2010) 238:233–46. 10.1111/j.1600-065X.2010.00952.x 20969596 PMC2988494

[22] Luetke-Eversloh M Cicek BB Siracusa F Thom JT Hamann A Frischbutter S. NK cells gain higher IFN-γ competence during terminal differentiation. Eur J Immunol. (2014) 44:2074–84. 10.1002/eji.201344072 24752800

[23] Luetke-Eversloh M Hammer Q Durek P Nordström K Gasparoni G Pink M. Human cytomegalovirus drives epigenetic imprinting of the IFNG locus in NKG2Chi natural killer cells. PLoS Pathog. (2014) 10:e1004441. 10.1371/journal.ppat.1004441 25329659 PMC4199780

[24] Foltz JA Moseman JE Thakkar A Chakravarti N Lee DA. TGFβ imprinting during activation promotes natural killer cell cytokine hypersecretion. Cancers. (2018) 10:E423. 10.3390/cancers10110423 30400618 PMC6267005

[25] Tang PM-K Zhou S Meng X-M Wang Q-M Li C-J Lian G-Y. Smad3 promotes cancer progression by inhibiting E4BP4-mediated NK cell development. Nat Commun. (2017) 8:14677. 10.1038/ncomms14677 28262747 PMC5343519

[26] Male V Nisoli I Kostrzewski T Allan DSJ Carlyle JR Lord GM. The transcription factor E4bp4/Nfil3 controls commitment to the NK lineage and directly regulates Eomes and Id2 expression. J Exp Med. (2014) 211:635–42. 10.1084/jem.20132398 24663216 PMC3978281

[27] van der Veeken J Zhong Y Sharma R Mazutis L Dao P Pe'er D. Natural genetic variation reveals key features of epigenetic and transcriptional memory in virus-specific CD8 T cells. Immunity. (2019) 50:1202–17.e7. 10.1016/j.immuni.2019.03.031 31027997 PMC7023907

[28] Assmann N O'Brien KL Donnelly RP Dyck L Zaiatz-Bittencourt V Loftus RM. Srebp-controlled glucose metabolism is essential for NK cell functional responses. Nat Immunol. (2017) 18:1197–206. 10.1038/ni.3838 28920951

[29] Loftus RM Assmann N Kedia-Mehta N O'Brien KL Garcia A Gillespie C. Amino acid-dependent cMyc expression is essential for NK cell metabolic and functional responses in mice. Nat Commun. (2018) 9:2341. 10.1038/s41467-018-04719-2 29904050 PMC6002377

[30] Zaiatz-Bittencourt V Finlay DK Gardiner CM. Canonical TGF-β signaling pathway represses human NK cell metabolism. J Immunol. (2018) 200:3934–41. 10.4049/jimmunol.1701461 29720425

[31] Gao Y Souza-Fonseca-Guimaraes F Bald T Ng SS Young A Ngiow SF. Tumor immunoevasion by the conversion of effector NK cells into type 1 innate lymphoid cells. Nat Immunol. (2017) 18:1004–15. 10.1038/ni.3800 28759001

[32] Cortez VS Ulland TK Cervantes-Barragan L Bando JK Robinette ML Wang Q. SMAD4 impedes the conversion of NK cells into ILC1-like cells by curtailing non-canonical TGF-β signaling. Nat Immunol. (2017) 18:995–1003. 10.1038/ni.3809 28759002 PMC5712491

[33] Fuchs A. ILC1s in tissue inflammation and infection. Front Immunol. (2016) 7:104. 10.3389/fimmu.2016.00104 27047491 PMC4801855

[34] Cella M Gamini R Sécca C Collins PL Zhao S Peng V Subsets of ILC3-ILC1-like cells generate a diversity spectrum of innate lymphoid cells in human mucosal tissues. Nat Immunol. (2019) 20:980–91. 10.1038/s41590-019-0425-y 31209406 PMC6685551

[35] Koipally J Renold A Kim J Georgopoulos K. Repression by Ikaros and Aiolos is mediated through histone deacetylase complexes. EMBO J. (1999) 18:3090–100. 10.1093/emboj/18.11.3090 10357820 PMC1171390

[36] Regis S Caliendo F Dondero A Bellora F Casu B Bottino C Main NK cell receptors and their ligands: regulation by microRNAs. Allergy. (2018) 2:98–112. 10.3934/Allergy.2018.2.98

[37] Zingoni A Molfetta R Fionda C Soriani A Paolini R Cippitelli M NKG2D and its ligands: “one for all, all for one.” Front Immunol. (2018) 9:476 10.3389/fimmu.2018.00476 29662484 PMC5890157

[38] Espinoza JL Takami A Yoshioka K Nakata K Sato T Kasahara Y. Human microRNA-1245 down-regulates the NKG2D receptor in natural killer cells and impairs NKG2D-mediated functions. Haematologica. (2012) 97:1295–303. 10.3324/haematol.2011.058529 22491735 PMC3436229

[39] Espinoza JL Nguyen VH Ichimura H Pham TTT Nguyen CH Pham TV. A functional polymorphism in the NKG2D gene modulates NK-cell cytotoxicity and is associated with susceptibility to Human Papilloma Virus-related cancers. Sci Rep. (2016) 6:39231. 10.1038/srep39231 27995954 PMC5172372

[40] Hayashi T Imai K Morishita Y Hayashi I Kusunoki Y Nakachi K. Identification of the NKG2D haplotypes associated with natural cytotoxic activity of peripheral blood lymphocytes and cancer immunosurveillance. Cancer Res. (2006) 66:563–70. 10.1158/0008-5472.CAN-05-2776 16397273

[41] Tomasello E Vivier E. KARAP/DAP12/TYROBP: three names and a multiplicity of biological functions. Eur J Immunol. (2005) 35:1670–7. 10.1002/eji.200425932 15884055

[42] Hoorweg K Peters CP Cornelissen F Aparicio-Domingo P Papazian N Kazemier G. Functional differences between human NKp44(-) and NKp44(+) RORC(+) innate lymphoid cells. Front Immunol. (2012) 3:72. 10.3389/fimmu.2012.00072 22566953 PMC3342004

[43] Donatelli SS Zhou J-M Gilvary DL Eksioglu EA Chen X Cress WD. TGF-β-inducible microRNA-183 silences tumor-associated natural killer cells. Proc Natl Acad Sci USA. (2014) 111:4203–8. 10.1073/pnas.1319269111 24586048 PMC3964044

[44] Trinh TL Kandell WM Donatelli SS Tu N Tejera MM Gilvary DL. Immune evasion by TGFβ-induced miR-183 repression of MICA/B expression in human lung tumor cells. OncoImmunology. (2019) 8:e1557372. 10.1080/2162402X.2018.1557372 30906652 PMC6422376

[45] Griffith JW Sokol CL Luster AD. Chemokines and chemokine receptors: positioning cells for host defense and immunity. Annu Rev Immunol. (2014) 32:659–702. 10.1146/annurev-immunol-032713-120145 24655300

[46] Sciumè G De Angelis G Benigni G Ponzetta A Morrone S Santoni A. CX3CR1 expression defines 2 KLRG1+ mouse NK-cell subsets with distinct functional properties and positioning in the bone marrow. Blood. (2011) 117:4467–75. 10.1182/blood-2010-07-297101 21364193

[47] Ponzetta A Sciumè G Benigni G Antonangeli F Morrone S Santoni A. CX3CR1 regulates the maintenance of KLRG1+ NK cells into the bone marrow by promoting their entry into circulation. J Immunol. (2013) 191:5684–94. 10.4049/jimmunol.1300090 24184559

[48] Regis S Caliendo F Dondero A Casu B Romano F Loiacono F. TGF-β1 downregulates the expression of CX3CR1 by inducing miR-27a-5p in primary human NK cells. Front Immunol. (2017) 8:868. 10.3389/fimmu.2017.00868 28791023 PMC5524732

[49] Neviani P Wise PM Murtadha M Liu CW Wu C-H Jong AY. Natural killer–derived exosomal miR-186 inhibits neuroblastoma growth and immune escape mechanisms. Cancer Res. (2019) 79:1151–64. 10.1158/0008-5472.CAN-18-0779 30541743 PMC6428417

[50] Berrien-Elliott MM Sun Y Neal C Ireland A Trissal MC Sullivan RP. MicroRNA-142 Is critical for the homeostasis and function of type 1 innate lymphoid cells. Immunity. (2019) 51:479–90.e6. 10.1016/j.immuni.2019.06.016 31402259 PMC6750984

[51] Xu D Han Q Hou Z Zhang C Zhang J. miR-146a negatively regulates NK cell functions via STAT1 signaling. Cell Mol Immunol. (2017) 14:712–20. 10.1038/cmi.2015.113 26996068 PMC5549603

[52] Paolini R Bernardini G Molfetta R Santoni A. NK cells and interferons. Cytokine Growth Factor Rev. (2015) 26:113–20. 10.1016/j.cytogfr.2014.11.003 25443799

[53] Tang PM-K Zhang Y-Y Lan H-Y. LncRNAs in TGF-β-driven tissue fibrosis. Non Coding RNA. (2018) 4:E26. 10.3390/ncrna4040026 30287731 PMC6315857

[54] Hao Y Baker D Ten Dijke P. TGF-β-mediated epithelial-mesenchymal transition and cancer metastasis. Int J Mol Sci. (2019) 20:E2767. 10.3390/ijms20112767 31195692 PMC6600375

[55] Tran HC Wan Z Sheard MA Sun J Jackson JR Malvar J. TGFβR1 blockade with Galunisertib (LY2157299) enhances anti-neuroblastoma activity of the anti-GD2 antibody dinutuximab (ch14.18) with natural killer cells. Clin Cancer Res. (2017) 23:804–13. 10.1158/1078-0432.CCR-16-1743 27756784 PMC5361893

[56] Wang Q-M Tang PM-K Lian G-Y Li C Li J Huang X-R. Enhanced cancer immunotherapy with Smad3-silenced NK-92 cells. Cancer Immunol Res. (2018) 6:965–77. 10.1158/2326-6066.CIR-17-0491 29915022

[57] Burga RA Yvon E Chorvinsky E Fernandes R Cruz CRY Bollard CM. Engineering the TGFβ receptor to enhance the therapeutic potential of natural killer cells as an immunotherapy for neuroblastoma. Clin Cancer Res. (2019) 25:4400–12. 10.1158/1078-0432.CCR-18-3183 31010834 PMC6635028

